# Association of “weekend warrior” and leisure time physical activity patterns with health-related physical fitness: a cross-sectional study

**DOI:** 10.1186/s12889-025-24711-y

**Published:** 2025-10-03

**Authors:** Stanley Sai-chuen HUI, Edwin Chun-yip CHIN, Jacky Ka-Wai CHAN, Ben Ping-Shing CHAN, James Ho-pong WAN, Sam Wing-Sum WONG

**Affiliations:** 1 Department of Sports Science and Physical Education, The Chinese University of Hong Kong, Hong Kong, China; 2 Department of Statistics, The Chinese University of Hong Kong, Sha Tin, Hong Kong China; 3 Physical Fitness Association of Hong Kong, Kowloon, Hong Kong China

**Keywords:** Weekend warrior, Exercise frequency, Health-related physical fitness

## Abstract

**Background:**

Few studies have examined the associations between “weekend warrior” activity patterns and health-related physical fitness.

**Purpose:**

This study investigated the associations between “weekend warrior” and leisure time physical activity patterns on health-related physical fitness.

**Methods:**

Data were extracted from the Hong Kong Territory-wide Physical Fitness Survey 2021. The sampling was based on the proportion of citizens’ demographic distribution (age, gender, employment status, working industries) indicated from the Hong Kong government’s 2021 population census statistics. Moderate-to-vigorous physical activity (MVPA) patterns were collected through questionnaires and categorized as follows: inactive (no MVPA), insufficiently active (< 150 min of MVPA per week), weekend warrior (≥ 150 min of MVPA on one or two days per week), and regularly active (≥ 150 min of MVPA on three or more days per week). All health-related physical fitness components were measured, including cardiorespiratory fitness (VO2max), muscular strength (handgrip strength), muscular endurance (plank), flexibility (sit and reach), and body composition (fat mass/fat-free mass).

**Results:**

A total of 5,091 adults aged 17–59 years were recruited. One hundred twenty-four test sessions were conducted across all administrative districts in Hong Kong. Compared with inactive individuals, regularly active individuals performed significantly better in all measured health-related fitness components. Weekend warriors also demonstrated superior fitness levels to their inactive counterparts, except in cardiorespiratory fitness. When considering only vigorous intensity, weekend warrior individuals still achieved better VO2max, handgrip strength, plank, sit and reach, and body composition than inactive individuals. Furthermore, those who were insufficiently active but engaged in at least three days of MVPA or who completed half of the recommended MVPA volume still achieved significantly better performance across all fitness components than the inactive individuals.

**Conclusion:**

Individuals who do not have time to be regularly active can still benefit in most health-related physical fitness components by performing the recommended volume of physical activity once or twice a week.

**Supplementary Information:**

The online version contains supplementary material available at 10.1186/s12889-025-24711-y.

## Introduction

The World Health Organization (WHO) recommends that adults engage in at least 150 min of moderate-to-vigorous physical activity (MVPA) per week, but does not specify the minimum frequency required to meet this target [1]. On the other hand, the American College of Sports Medicine (ACSM) suggests distributing this activity across at least five days per week [2]. However, adhering to such a schedule can be challenging, particularly for adults with demanding weekday commitments who may only have time for exercise on weekends. Research has identified “lack of time” as a primary barrier to regular physical activity [3], highlighting the need to explore lower-frequency schedules that still achieve the recommended MVPA volume. While the current WHO guidelines emphasize that MVPA can increase physical fitness among children and adolescents (aged 5 to 17) [1], evidence on how achieving 150 min of MVPA weekly impacts physical fitness in adults is lacking. Physical fitness is a critical indicator of overall health, linked to various health outcomes such as all-cause mortality [4], cardiovascular disease mortality [4], cancer mortality [4], decline in activities of daily living [5], cognitive decline [5], and increased risk of injury [6]. Moreover, increasing physical activity is one of the most effective ways to improve all health-related fitness components [7–10]. Examining these fitness components can help determine whether reducing the frequency of activity (i.e., sessions per week) can still deliver comparable health and fitness benefits.

The “weekend warrior” activity pattern refers to individuals who accumulate the recommended amount of aerobic physical activity over 1 or 2 days of the week [11]. Epidemiological studies extensively show the various health outcomes associated with this activity pattern, including reduced all-cause mortality [12, 13], cardiovascular disease mortality [12, 13], cancer mortality [12], a lower incidence of cardiovascular disease [14], metabolic syndrome [15], psychological distress [16], and depression symptoms [17]. As for the impact of the weekend warrior activity pattern on physical fitness, a randomized controlled trial demonstrated that two consecutive days of aerobic training weekly resulted in similar improvements in cardiorespiratory fitness compared with the same amount of training over five days per week [18]. However, the sample size of this study was relatively small (~ 13 participants per group). Additional data is warranted to confirm this finding. With respect to body composition, a cross-sectional study revealed that both the weekend warrior and regular active activity patterns have lower abdominal and overall adiposity than the inactive pattern [19]. Another research also showed that weekend warrior activity pattern could decrease abnormal waist circumference and visceral adiposity index among adults [15, 20]. Nevertheless, as body composition encompasses muscle, fat, bone, and other vital components [21], studies focusing solely on fat tissue are insufficient to fully assess the impact of weekend warrior pattern on body composition. Future research must incorporate measures of fat-free mass to provide the full picture of weekend warriors’ adaptation in body composition. Although the abovementioned studies examined the effect of weekend warrior activity patterns on cardiorespiratory fitness and body fatness, its impact on all health-related physical fitness components remains not sufficiently understood. Future research examining all health-related fitness components is warranted to deepen our understanding for improving physical fitness in time-constrained adults.

To address these research gaps, the primary objective of this study was to analyze the association between the weekend warrior activity pattern and all health-related physical fitness components (cardiorespiratory fitness, muscular strength, muscular endurance, flexibility, and body composition). Given that the weekend warrior pattern may reduce the number of weekly sessions but cannot lower the total time commitment, the secondary objective was to investigate whether insufficiently active individuals (i.e., < 150 min of MVPA per week) with only 1 to 2 sessions, or those performing half the recommended activity volume (75 min of MVPA weekly), can still yield comparable or slightly better health-related physical fitness than inactivity. Lastly, this study aimed to further investigate the impact of vigorous-intensity weekend warrior activities on these fitness components. By addressing these objectives, the findings of this study could inform public health strategies, particularly for adults with limited time for physical activity, by highlighting the potential benefits of lower-frequency activity patterns like the weekend warrior.

## Methods

### Study setting, design, and participants

**Fig. 1:**
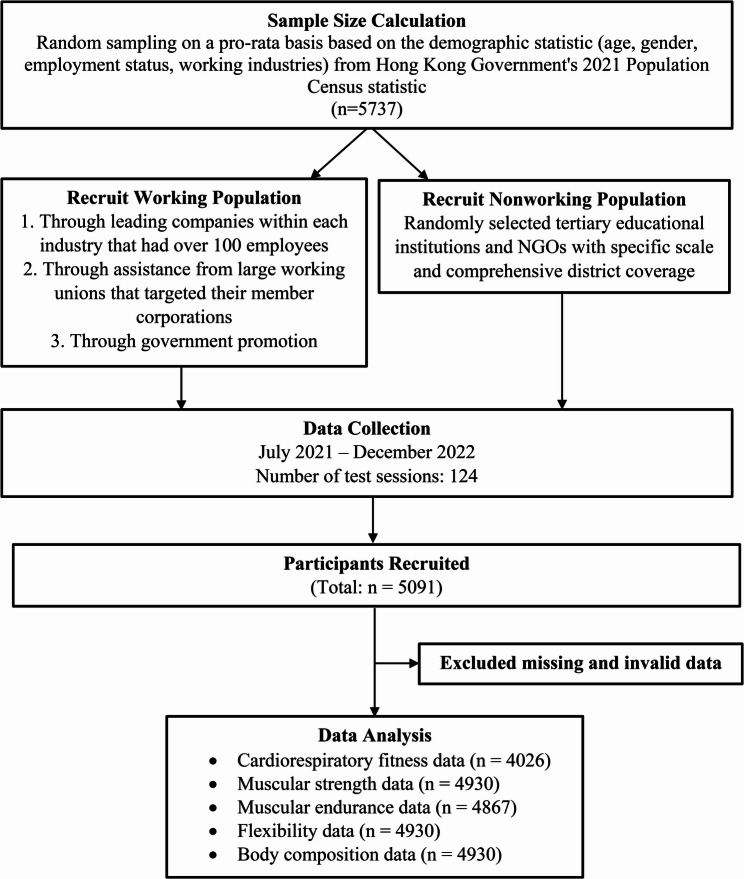
The recruitment and gradual selection process of participants

Given that the weekend warrior activity strategy aims to minimize weekly physical activity commitments, adults aged 17–59 years were selected from the Hong Kong Territory-wide Physical Fitness Survey 2021 database [22], as this age group often faces time constraints that limit their capacity to engage in regular physical activity. The recruitment and gradual selection process of the participants is displayed in Fig. 1. The Hong Kong Territory-wide Physical Fitness Survey 2021 is a cross-sectional city-wide community fitness survey conducted throughout Hong Kong. The data collection took place from July 2021 to December 2022. The survey was conducted by the Leisure and Cultural Services Department of the Hong Kong Government via face-to-face questionnaires and physical fitness assessments. Detailed information on the survey design, participant sampling, and data collection procedures has been previously published [22]. Participants who met the following criteria were excluded: physical maldevelopment, handicaps, pregnancy, or hospitalization for more than three consecutive days due to sickness or injury in the last three months. This study adhered to the reporting guideline of the Strengthening the Reporting of Observational Studies in Epidemiology (STROBE) [23].

### Study size

The target sample size in this study was 5,737 adults. The details of the sampling methods have been described elsewhere [22]. In brief, the sample size was determined on the basis of the age and gender distributions provided by the Hong Kong Government’s 2021 Population Census. Data collection involved various corporations, government and non-governmental organizations, and large-scale working unions. The sampling process covered all working industries and employment statuses for different age groups. For the working population, companies and federations were randomly selected on the basis of the economic categories provided by the Census & Statistics Department of the Hong Kong Government. Arrangements were then made with the participants who agreed to participate in this survey. For the nonworking population, participants were randomly selected through tertiary institutions, District Social Welfare Offices, and non-government organizations with a specific scale and comprehensive district coverage. The sample size calculation also considered the proportion of the working and nonworking population using data from the 2021 Population Census. Citizens from all 18 districts in Hong Kong were included in this survey.

### Assessment of physical activity

We categorized physical activity into four patterns: inactive, insufficiently active, weekend warrior, and regularly active. The inactive pattern refers to individuals who do not engage in MVPA. The insufficiently active pattern is characterized by individuals participating in MVPA for less than 150 min per week. The weekend warrior pattern refers to individuals who engage in at least 150 min per week of MVPA but only from 1 to 2 sessions weekly. Finally, the regularly active pattern is characterized by individuals who engage in at least 150 min per week of MVPA from at least three sessions weekly.

We used four questions to gather information on the frequency and weekly duration of MVPA and vigorous-intensity physical activity (VPA). The complete questionnaire is provided in Supplementary 1. The first question concerned the frequency with which the participants engaged in at least 10 min of MVPA and VPA. If participants reported engaging in MVPA and VPA, we then inquired about the amount of time they spent in these activities per week. To ensure clarity and understanding, we provided examples of MVPA and VPA to the participants. Additionally, we utilized the Rating of Perceived Exertion scale, which ranges from 0 to 10, to help participants better identify the intensity levels of the activities. We defined vigorous intensity as a Rating of Perceived Exertion (RPE) rating of 8 to 9 and moderate intensity as an RPE rating of 4 to 7. Trained and certified fitness assessors from the Physical Fitness Association of Hong Kong, China, conducted the interviews and administered the questionnaire.

### Assessment of health-related fitness

All fitness tests were conducted by trained and certified fitness assessors from the Physical Fitness Association of Hong Kong, China.

**Cardiorespiratory fitness.** The YMCA 3-minute step test was used to evaluate cardiorespiratory fitness. The testing procedure followed the YMCA Fitness Testing and Assessment Manual [24]. A metronome was set at 96 beats per minute. The participants stepped up and down on a 12-inch high box. Immediately afterward, the submaximal heart rate was measured within five seconds at the end of the exercise with a fingertip oximeter. The estimated maximal oxygen consumption (VO2max) from the YMCA step test was 25.8 [25]. VO2max was estimated via the following calculation [26].

Male:$$\:\:\text{V}\text{O}2\text{m}\text{a}\text{x}=25.8\times\:\frac{\left(220-\text{a}\text{g}\text{e}\right)-61}{\text{I}\text{m}\text{m}\text{e}\text{d}\text{i}\text{a}\text{t}\text{e}\:\text{H}\text{e}\text{a}\text{r}\text{t}\:\text{R}\text{a}\text{t}\text{e}\:\text{a}\text{f}\text{t}\text{e}\text{r}\:\text{S}\text{t}\text{e}\text{p}\:\text{T}\text{e}\text{s}\text{t}-61}$$

Female:$$\:\:\text{V}\text{O}2\text{m}\text{a}\text{x}=25.8\:\:\times\:\frac{\left(220-\text{a}\text{g}\text{e}\right)-73}{\text{I}\text{m}\text{m}\text{e}\text{d}\text{i}\text{a}\text{t}\text{e}\:\text{H}\text{e}\text{a}\text{r}\text{t}\:\text{R}\text{a}\text{t}\text{e}\:\text{a}\text{f}\text{t}\text{e}\text{r}\:\text{S}\text{t}\text{e}\text{p}\:\text{T}\text{e}\text{s}\text{t}-73}$$

**Muscular Strength** was measured via a handgrip strength test with a hand-held digital isometric dynamometer (T.K.K.5401, Takei, Japan). The participants stood with their feet shoulder-width apart, and their Hands hung down naturally on both sides of the body. The participants squeezed the dynamometer as hard as possible for 2–3 s with verbal encouragement given by the tester. Three trials were conducted for each hand, and the maximum scores for the left and right hands were combined and recorded for analysis.

**Muscular Endurance** was examined via the plank test. The participants started the test by holding a prone bridge position with their forearms and feet touching the ground. Throughout the test, the participants were required to keep their elbows directly beneath their shoulders, with their forearms and fingers extended straight ahead. The participants needed to maintain a straight line from head to heel, facing the ground. If a participant was unable to sustain proper body alignment, reminders were given. However, if the participant continued to struggle and could not maintain the desired straight line, the test was terminated, and the time spent in seconds was recorded.

**Flexibility** was assessed via the sit-and-reach test. The participants were instructed to sit on the mat and remove their shoes. The sit-and-reach box was placed on the mat and against the wall. The participants fully extended both legs with their heels touching the bottom of the box. They overlapped both hands and reached forward toward the extended leg three times. The maximum distance (cm) was recorded for analysis.

**Body Composition** was measured by a multifrequency bioelectrical impedance analyzer (MC780, Tanita). The participants were instructed to step on the footpads of the analyzer with bare feet and to hold the handgrip with both hands. To ensure accuracy and minimize any potential interference, the participants were asked to wear minimal clothing and clean their feet with alcohol before the test. A previous study demonstrated a significant correlation between the body fat percentage measured by the Tanita MC780 and the gold standard measurement of body fat percentage via dual-energy X-ray absorptiometry (*r* = 0.852, *p* < 0.001) [27]. The intraclass correlation coefficient of Tanita MC780 in assessing body adiposity is 0.84, which further supports the reliability of the Tanita MC780 in assessing body fat percentage [27]. The fat mass-to-fat-free mass ratio [28] was used to evaluate body composition. A lower ratio indicates better body composition.

### Covariates

The trained and certified fitness assessors collected the covariate information from the participants. These included sex, age, education level, household income, smoking status, and sleep quality. Education levels were categorized into three groups: primary school or below, secondary school, and post-secondary education. Household incomes (in Hong Kong Dollar) were divided into eight categories: (1) 9,999, (2) 10,000–19,999, (3) 20,000–29,999, (4) 30,000–39,999, (5) 40,000–49,999, (6) 50,000–59,999, (7) 60,000–99,999, and (8) 100,000 or above. The participants were asked about their smoking status, which was classified into three categories: never smoked, former smoker (quit smoking within the past six months), and current smoker. To assess sleep quality, participants were asked to rate their sleep quality on a 5-point scale (1: very good, 2: good, 3: average, 4: bad, 5: very bad) over the past month. Additionally, the fitness assessors measured resting blood pressure to determine whether the participants had hypertension. Hypertension was defined according to the 2020 International Society of Hypertension Global Hypertension Practice Guidelines (systolic blood pressure ≥ 140 mmHg and diastolic blood pressure ≥ 90 mmHg) [29]. Abdominal obesity was determined via the International Diabetes Federation’s criteria for the Asian population, defined as a waist circumference of ≥ 90 cm for men and ≥ 80 cm for women [30]. Waist circumference was measured with a Gulick tape, following WHO guidelines [31]. General obesity was classified as a BMI of ≥ 25 kg/m² on the basis of the WHO criteria for Asian adults [32].

### Statistical analysis

The generalized linear model (GLM) was used to evaluate the association between physical activity variables and fitness variables. This approach was chosen because the physical activity data are categorical, while the physical fitness data are continuous. Additionally, GLMs provide flexibility in assuming different distributions for the dependent variables, allowing for better modeling of the data. Four association tests were conducted involving five health-related fitness components (cardiovascular fitness, muscular strength, muscular endurance, flexibility, and body composition) and four physical activity variables: [1] MVPA patterns [2], MVPA patterns with distinctions in the frequency of the insufficiently active group [3], weekly duration of MVPA, and [4] vigorous physical activity patterns. Thus, a total of 20 GLMs were used in this study. Each GLM analysis consisted of three models for the adjustment of covariates. Model 1 did not involve any covariates. In Model 2, we included adjustments for demographic covariates (i.e., gender, age, education, and household income). Finally, Model 3 included demographic covariates, general obesity status, central obesity status, smoking status, hypertension, and sleep quality. The Akaike’s Information Criterion (AIC) was used to compare the goodness of fit among Models 1, 2, and 3. A smaller AIC value is considered to indicate a better fit. The distribution of each physical fitness variable was assessed during the exploratory analysis phase to determine the most appropriate distribution for use in the GLMs.

The assumption of missing completely at random (MCAR) was applied to the dataset because all missing data were caused by technical errors, such as assessors omitted to enter the data, assessors inputting extremely high or unreasonable values, or equipment failure. As a result, GLM analyses were not biased by removing missing data in the GLM analysis [33]. The inactive group served as the reference group. The GLM results are presented as beta coefficients (β) and 99% confidence intervals (CIs). All the statistical analyses were conducted via SPSS software (version 27; IBM Corp). Statistical significance was determined by a p-value of less than 0.01.

## Results

### Characteristics of study participants

A total of 5,091 participants were recruited for this survey. The data were collected between July 2021 and December 2022. After removing the invalid and missing data, 4,026 VO2max data, 4,930 Handgrip data, 4,867 plank data, 4,930 sit-and-reach data, and 4,930 fat mass/fat-free mass ratio data points were included in the data analysis. The characteristics of the participants and the range of quartiles for each fitness parameter are presented in Table 1. VO2max and handgrip strength data were modeled using the Gamma distribution, plank duration and sit-and-reach flexibility data were analyzed with the linear distribution, and the fat mass/fat-free mass ratio data were fitted using the inverse Gaussian distribution.

**Table 1:** Participant characteristics

	Inactive	Insufficiently Active	Weekend Warrior	Regularly Active
Gender, *n* (%)				
Female	604 (69.8)	1441 (61.8)	223 (57.9)	711 (52.7)
Male	261 (30.2)	889 (38.2)	162 (42.1)	639 (47.3)
Age Group, n (%)				
17–19	10 (1.2)	57 (2.4)	10 (2.6)	68 (5.0)
20–39	284 (32.8)	960 (41.2)	180 (46.8)	537 (39.8)
40–59	571 (66.0)	1313 (56.4)	195 (50.6)	745 (55.2)
Waist Circumference Conditions, n (%)				
Normal	595 (68.9)	1745 (75.0)	296 (77.1)	1074 (79.6)
Abdominal obesity	268 (31.1)	581 (25.0)	88 (22.9)	275 (20.4)
BMI Conditions, n (%)				
Underweight (BMI < 18.5)	59 (6.8)	155 (6.7)	21 (5.5)	46 (3.4)
Normal (BMI 18.5–22.9)	399 (46.1)	1142 (49.0)	195 (50.6)	680 (50.4)
Overweight (BMI 23-24.9)	171 (19.8)	455 (19.5)	79 (20.5)	285 (21.1)
Obese I (BMI 25-29.9)	187 (21.6)	465 (20.0)	73 (19.0)	300 (22.2)
Obese II (BMI ≥ 30.0)	49 (5.7)	113 (4.8)	17 (4.4)	39 (2.9)
Education Level, n (%)				
Primary school or below	25 (2.9)	34 (1.5)	4 (1.0)	23 (1.7)
Secondary school	364 (42.1)	701 (30.5)	99 (26.0)	437 (32.8)
Postsecondary	463 (53.5)	1562 (68.0)	278 (73.0)	871 (65.4)
Refused to answer	13 (1.5)	33 (1.4)	4 (1.0)	19 (1.4)
Household Income, n (%)				
$9,999 or below	94 (10.9)	251 (10.8)	30 (7.8)	144 (10.7)
$10,000–19,999	154 (17.8)	350 (15.0)	49 (12.7)	193 (14.3)
$20,000–29,999	157 (18.2)	396 (17.0)	67 (17.4)	199 (14.7)
$30,000–39,999	97 (11.2)	314 (13.5)	58 (15.1)	155 (11.5)
$40,000–59,999	71 (8.2)	205 (8.8)	28 (7.3)	104 (7.7)
$60,000–99,999	86 (9.9)	284 (12.2)	55 (14.3)	141 (10.4)
$100,000 or above	34 (3.9)	140 (6.0)	27 (7.0)	89 (6.6)
Uncertain or refused to answer	172 (19.9)	390 (16.7)	71 (18.4)	325 (24.1)
Smoking Status, n (%)				
Never	777 (89.8)	2121 (91.0)	345 (89.6)	1206 (89.3)
Former Smokers	23 (2.7)	67 (2.9)	21 (5.5)	66 (4.9)
Current Smokers	65 (7.5)	142 (6.1)	19 (4.9)	78 (5.8)
Hypertension, n (%)				
Normal	722 (83.7)	1900 (82.0)	313 (81.5)	1086 (80.7)
Hypertension	141 (16.3)	418 (18.0)	71 (18.5)	259 (19.3)
VO2max, range of quartile, ml/kg/min				
First quartile	26.0–31.0	26.0–32.0	26.0–33.0	26.0–33.0
Second quartile	31.0–36.0	32.0–37.0	33.0–37.0	33.0–39.0
Third quartile	36.0–41.0	37.0–42.0	37.0–43.0	39.0–45.0
Fourth quartile	41.0–61.0	42.0–68.0	43.0–61.0	45.0–69.0
Handgrip strength, range of quartile, kg				
First quartile	22.0–45.0	21.0–47.0	29.0–49.0	24.0–49.0
Second quartile	45.0–52.0	47.0–55.0	49.0–57.0	49.0–60.0
Third quartile	52.0–66.0	55.0–70.0	57.0-75.3	60.0–77.0
Fourth quartile	66.0-114.0	70.0-122.0	75.0-114.0	77.0-119.0
Plank, range of quartile, s				
First quartile	0.0–43.0	1.0–53.0	12.0-56.8	4.0–63.0
Second quartile	43.0-65.5	53.0–75.0	56.8–88.5	63.0–93.0
Third quartile	65.5–100.0	75.0-117.3	88.5-123.3	93.0-134.0
Fourth quartile	100.0-480.0	177.3–588.0	123.3–510.0	134.0-634.0
Sit and reach, range of quartile, cm				
First quartile	0.0–18.0	0.0–20.0	1.0–22.0	1.0–22.0
Second quartile	18.0–25.0	20.0–27.0	22.0–28.0	22.0–30.0
Third quartile	25.0–33.0	27.0–34.0	28.0–35.0	30.0–36.0
Fourth quartile	33.0–53.0	34.0–53.0	35.0–48.0	36.0–53.0
Fat mass/fat-free mass, range of quartile, ratio				
First quartile	0.06–0.31	0.03–0.28	0.05–0.25	0.03–0.26
Second quartile	0.31–0.40	0.28–0.38	0.25–0.36	0.26–0.35
Third quartile	0.40–0.50	0.38–0.48	0.36–0.46	0.35–0.45
Fourth quartile	0.50–1.38	0.48–1.35	0.46–0.84	0.45–1.32

Notes:

Inactive: No engagement in MVPA physical activities

Insufficiently active: Engaged in less than 150 min of MVPA per week

Weekend warrior: Engaged in at least 150 min of MVPA per week, but only from 1 or 2 sessions weekly

Regularly active: Engaged in at least 150 min per week of MVPA from 3 or more sessions weekly

Abdominal obesity: waist circumference ≥ 90 cm for men and ≥ 80 cm for women

BMI conditions were classified by the WHO specifically for Asian adults

Hypertension: Systolic blood pressure ≥ 140 mmHg or diastolic blood pressure ≥ 90 mmHg

The currency used to quantify household income is the Hong Kong dollar

### Associations between MVPA patterns and fitness

Table 2 presents the associations between MVPA patterns and health-related fitness. A hierarchical order of beta coefficients was observed across all health-related fitness levels, progressing from the inactive group to the regularly active groups. The adjusted models (Models 2 and 3) demonstrated that regularly active individuals had significantly better cardiovascular fitness than inactive individuals. However, the weekend warrior group did not show a significant difference in cardiovascular fitness when compared with the inactive group. The adjusted models also indicated that regularly active and weekend warrior groups had significantly greater muscular strength than their inactive counterparts. Furthermore, all three activity patterns (insufficiently active, weekend warrior, and regularly active) had significantly better muscular endurance and flexibility than the inactive group across all models of GLM. With respect to body composition, the fully adjusted GLM analysis revealed that all three activity patterns (insufficiently active, weekend warriors, and regularly active) had significantly lower fat mass-to-fat-free mass ratio than the inactive group.

**Table 2:** The association between Moderate-to-vigorous physical activity patterns and Health-related physical fitness

			β (99%CI)		
	**Cardiorespiratory fitness** (VO2max)	**Muscular Strength** (Handgrip)	**Muscular Endurance** (Plank)	**Flexibility** (Sit and Reach)	**Body Composition** (Fat Mass/Fat-free mass)
Model 1					
Inactive	0 [Reference]	0 [Reference]	0 [Reference]	0 [Reference]	0 [Reference]
Insufficiently active	1.05 (0.23, 1.86)*	3.16 (1.56, 4.76)*	12.42 (6.04, 18.80)*	1.24 (0.23, 2.25)*	−0.03 (−0.06, −0.01)*
Insufficiently active, 1 or 2 days	0.82 (−0.04, 1.68)	2.30 (0.60, 3.99)*	9.57 (2.82, 16.33)*	1.08 (0.01, 2.15)*	−0.03 (−0.05, −0.01)*
Insufficiently active, ≥ 3 days	1.55 (0.51, 2.60)*	5.02 (2.94, 7.10)*	18.60 (10.59, 26.61)*	1.59 (0.32, 2.86)*	−0.05 (−0.07, −0.02)*
Weekend warrior	1.86 (0.58, 3.14)*	5.99 (3.38, 8.60)*	19.95 (10.19, 29.72)*	2.73 (1.18, 4.28)*	−0.06 (−0.10, −0.03)*
Regularly active	3.21 (2.28, 4.14)*	8.85 (7.02, 10.69)*	35.54 (28.57, 42.52)*	3.62 (2.52, 4.72)*	−0.08 (−0.10, −0.06)*
Model 2					
Inactive	0 [Reference]	0 [Reference]	0 [Reference]	0 [Reference]	0 [Reference]
Insufficiently active	0.70 (−0.10, 1.49)	0.92 (−0.04, 1.89)	8.96 (2.81, 15.12)*	1.67 (0.73, 2.61)*	−0.02 (−0.03, −0.00)
Insufficiently active, 1 or 2 days	0.35 (−0.48, 1.19)	0.52 (−0.51, 1.54)	6.39 (−0.13, 12.92)	1.33 (0.33, 2.33)*	−0.01 (−0.03, 0.01)
Insufficiently active, ≥ 3 days	1.44 (0.43, 2.45)*	1.80 (0.56, 3.05)*	14.42 (6.72, 22.11)*	2.38 (1.20, 3.56)*	−0.03 (−0.05, −0.01)*
Weekend warrior	1.27 (0.03, 2.51)*	2.32 (0.76, 3.89)*	15.24 (5.82, 24.65)*	3.33 (1.88, 4.78)*	−0.03 (−0.06, −0.01)*
Regularly active	2.94 (2.04, 3.83)*	3.63 (2.53, 4.74)*	29.91 (23.15, 36.67)*	4.62 (3.59, 5.66)*	−0.04 (−0.06, −0.03)*
Model 3					
Inactive	0 [Reference]	0 [Reference]	0 [Reference]	0 [Reference]	0 [Reference]
Insufficiently active	0.65 (−0.13, 1.44)	1.04 (0.08, 1.99)*	7.28 (1.30, 13.26)*	1.46 (0.53, 2.39)*	−0.01 (−0.02, 0.00)*
Insufficiently active, 1 or 2 days	0.32 (−0.51, 1.15)	0.66 (−0.36, 1.67)	5.00 (−1.34, 11.34)	1.17 (0.18, 2.16)*	−0.01 (−0.02, 0.00)
Insufficiently active, ≥ 3 days	1.38 (0.38, 2.38)*	1.87 (0.63, 3.10)*	12.10 (4.62, 19.58)*	2.07 (0.90, 3.23)*	−0.02 (−0.03, −0.00)*
Weekend warrior	1.22 (−0.00, 2.45)	2.40 (0.85, 3.95)*	13.37 (4.21, 22.53)*	3.09 (1.66, 4.52)*	−0.02 (−0.04, −0.01)*
Regularly active	2.89 (1.99, 3.78)*	3.75 (2.66, 4.85)*	26.89 (20.31, 33.47)*	4.26 (3.24, 5.29)*	−0.03 (−0.05, −0.02)*
Δ AIC					
Model 2 − 1	− 257	−4520	−397	−727	−1635
Model 3 − 2	−180	−335	−582	−350	−1802

Model 1 was the univariate model in which no covariates were adjusted

Model 2 was adjusted for demographic covariates, including sex, age, education level, and household income level

Model 3 was additionally adjusted for general obesity status, central obesity status, hypertension status, and smoking status

* Indicates statistical significance (*p* < 0.01)

Furthermore, we have distinguished the physical activity frequencies of the insufficiently active group. The insufficiently active individuals who engaged in at least three days of physical activity per week had better performance across all health-related fitness components than the inactive individuals, regardless of covariate adjustment. However, the fully adjusted GLMs revealed that insufficiently active individuals who engaged in less than three days of MVPA/week did not have superior cardiovascular fitness, muscular strength, muscular endurance, or body composition compared with inactive individuals.

### Associations between weekly duration of MVPA and fitness

Table 3 illustrates the association between the weekly duration of MVPA and fitness. A hierarchical order of beta coefficients from inactive to regular active groups was observed across all fitness components. The adjusted GLM analyses revealed that even engaging in at least half of the MVPA weekly volume (75–149.9 min) was associated with significantly better performance in all health-related fitness components than being physically inactive.

**Table 3:** The association between weekly duration of MVPA and Health-related physical fitness

			β (99%CI)		
	**Cardiorespiratory fitness** (VO2max)	**Muscular Strength** (Handgrip)	**Muscular Endurance** (Plank)	**Flexibility** (Sit and Reach)	**Body Composition** (Fat Mass/Fat-free mass)
Model 1					
None	0 [Reference]	0 [Reference]	0 [Reference]	0 [Reference]	0 [Reference]
10–74.9 min	0.69 (−0.19, 1.57)	2.28 (0.56, 4.00)*	7.05 (0.19, 13.92)*	1.02 (−0.07, 2.10)	−0.03 (−0.05, −0.00)*
75–149.9 min	1.63 (0.64, 2.62)*	4.60 (2.63, 6.57)*	21.25 (13.61, 28.89)*	1.61 (0.40, 2.82)*	−0.05 (−0.07, −0.02)*
150–299.9 min	2.50 (1.51, 3.49)*	6.34 (4.37, 8.31)*	28.28 (20.74, 35.82)*	2.75 (1.56, 3.94)*	−0.07 (−0.09, −0.04)*
≥ 300 min	3.39 (2.32, 4.46)*	10.38 (8.26, 12.51)*	36.43 (28.61, 44.24)*	4.20 (2.96, 5.44)*	−0.09 (−0.11, −0.06)*
Model 2					
None	0 [Reference]	0 [Reference]	0 [Reference]	0 [Reference]	0 [Reference]
10–74.9 min	0.25 (−0.60, 1.10)	0.56 (−0.48, 1.60)	4.78 (−1.83, 11.40)	1.28 (0.27, 2.29)*	−0.01 (−0.03, 0.01)
75–149.9 min	1.43 (0.47, 2.39)*	1.53 (0.35, 2.71)*	15.98 (8.60, 23.36)*	2.32 (1.19, 3.44)*	−0.03 (−0.04, −0.01)*
150–299.9 min	2.04 (1.08, 3.01)*	2.21 (1.03, 3.40)*	23.04 (15.74, 30.35)*	3.52 (2.41, 4.64)*	−0.04 (−0.06, −0.02)*
≥ 300 min	3.18 (2.15, 4.21)*	4.69 (3.41, 5.97)*	30.92 (23.35, 38.48)*	5.29 (4.13, 6.45)*	−0.05 (−0.07, −0.03)*
Model 3					
None	0 [Reference]	0 [Reference]	0 [Reference]	0 [Reference]	0 [Reference]
10–74.9 min	0.20 (−0.65, 1.04)	0.72 (−0.31, 1.75)	3.13 (−3.30, 9.55)	1.08 (0.08, 2.08)*	−0.01 (−0.02, 0.01)
75–149.9 min	1.40 (0.45, 2.35)*	1.57 (0.40, 2.74)*	14.22 (7.05, 21.39)*	2.08 (0.97, 3.20)*	−0.02 (−0.03, −0.01)*
150–299.9 min	1.96 (1.01, 2.92)*	2.32 (1.15, 3.49)*	19.88 (12.77, 27.00)*	3.16 (2.05, 4.27)*	−0.02 (−0.04, −0.01)*
≥ 300 min	3.16 (2.14, 4.18)*	4.80 (3.53, 6.06)*	28.61 (21.26, 35.96)*	4.99 (3.84, 6.13)*	−0.04 (−0.05, −0.03)*
Δ AIC					
Model 2 − 1	−261	−4520	−389	−730	−1633
Model 3 − 2	−182	−334	−589	−351	−1812

Model 1 was the univariate model in which no covariates were adjusted

Model 2 was adjusted for demographic covariates, including sex, age, education level, and household income level

Model 3 was additionally adjusted for general obesity status, central obesity status, hypertension status, and smoking status

* Indicates statistical significance (*p* < 0.01)

### Associations between VPA patterns and fitness

Table 4 shows the associations between VPA patterns and health-related fitness. The fully adjusted GLM analyses (Model 3) revealed that individuals performing at least 75 min of VPA weekly, whether following a weekend warrior or regularly active pattern, had significantly better performance across all health-related fitness components than the inactive group.

**Table 4:** The association between vigorous physical activity patterns and Health-related physical fitness

			β (99%CI)		
	**Cardiorespiratory fitness** (VO2max)	**Muscular Strength** (Handgrip)	**Muscular Endurance** (Plank)	**Flexibility** (Sit and Reach)	**Body Composition** (Fat Mass/Fat-free mass)
Model 1					
Inactive	0 [Reference]	0 [Reference]	0 [Reference]	0 [Reference]	0 [Reference]
Insufficiently active	1.06 (0.36, 1.76)*	3.87 (2.46, 5.27)*	8.72 (3.24, 14.20)*	0.37 (−0.51, 1.24)	−0.03 (−0.05, −0.02)*
Weekend warrior	2.49 (1.50, 3.48)*	7.39 (5.35, 9.42)*	24.79 (17.33, 32.26)*	1.59 (0.39, 2.78)*	−0.06 (−0.09, −0.04)*
Regularly active	4.03 (3.14, 4.91)*	11.14 (9.37, 12.91)*	37.66 (31.37, 43.95)*	2.80 (1.79, 3.81)*	−0.10 (−0.12, −0.08)*
Model 2					
Inactive	0 [Reference]	0 [Reference]	0 [Reference]	0 [Reference]	0 [Reference]
Insufficiently active	0.57 (−0.12, 1.26)	0.60 (−0.27, 1.47)	5.30 (−0.06, 10.66)*	1.12 (0.26, 1.91)*	−0.01 (−0.02, 0.00)
Weekend warrior	1.65 (0.68, 2.63)*	1.52 (0.26, 2.77)*	19.41 (12.08, 26.74)*	2.81 (1.67, 3.94)*	−0.02 (−0.04, 0.00)*
Regularly active	3.53 (2.66, 4.40)*	3.48 (2.39, 4.57)*	31.00 (24.78, 37.21)*	4.45 (3.49, 5.41)*	−0.04 (−0.06, −0.03)*
Model 3					
Inactive	0 [Reference]	0 [Reference]	0 [Reference]	0 [Reference]	0 [Reference]
Insufficiently active	0.53 (−0.16, 1.22)	0.70 (−0.16, 1.56)	4.12 (−1.09, 9.32)	0.99 (0.18, 1.81)*	−0.00 (−0.01, 0.01)
Weekend warrior	1.61 (0.64, 2.58)*	1.50 (0.26, 2.74)*	18.27 (11.16, 25.37)*	2.66 (1.54, 3.77)*	−0.01 (−0.02, 0.00)*
Regularly active	3.41 (2.55, 4.28)*	3.60 (2.52, 4.69)*	28.23 (22.17, 34.28)*	4.10 (3.15, 5.05)*	−0.03 (−0.04, −0.02)*
Δ AIC					
Model 2 − 1	−226	−4355	−362	−756	−1551
Model 3 − 2	−175	−336	−581	−347	−1790

Model 1 was the univariate model in which no covariates were adjusted

Model 2 was adjusted for demographic covariates, including sex, age, education level, and household income level

Model 3 was additionally adjusted for general obesity status, central obesity status, hypertension status, and smoking status

* Indicates statistical significance (*p* < 0.01)

## Discussion

To the best of our knowledge, this is the first study to examine the associations of weekend warrior activity pattern with health-related physical fitness. Our study has several important findings. First, individuals following a weekend warrior MVPA pattern outperformed inactive individuals in most health-related fitness components, with the exception of cardiovascular fitness. Second, engaging in at least three MVPA sessions per week, even without meeting the WHO’s recommended 150 min, benefited all fitness components. Third, even half of the recommended MVPA dose can benefit all health-related fitness components. Finally, when considering only vigorous intensity, both weekend warriors and regularly active individuals achieved better performance across all fitness components than inactive individuals.

### Association between the weekend warrior activity pattern and all health-related physical fitness components (the primary study’s objective)

To develop or maintain cardiorespiratory fitness, the American College of Sports Medicine (ACSM) position standard recommends that all adults engage in 150 min of moderate-intensity cardiorespiratory exercise for at least five days per week [2]. The results of the present study are aligned with the ACSM position stand. We found that only the regularly active individuals had significantly better cardiorespiratory fitness than the inactive individuals, while the weekend warrior group did not exhibit significantly greater VO2max than inactive group. However, it is important to note that the VO2max difference between weekend warrior and inactive group was very close to the statistical significance (*p* = 0.01) in the fully adjusted GLM analysis. Given that the total weekly activity volume was equivalent between the weekend warrior and regular active patterns, both physical activity patterns should have similar VO2max values. For this reason, we speculate that the weekend warrior activity pattern might show a significantly superior VO2max over inactivity if the sample size was increased. This speculation is supported by our previous randomized controlled trial, which revealed that aerobic training with a weekend warrior approach could improve VO2max [18]. However, this trial had a relatively small sample size (~ 10 participants per group), limiting the strength of its conclusions regarding the effectiveness of the weekend warrior approach on cardiovascular fitness. Similarly, the final analyzed VO2max data (*n* = 4,026) in present study represented only 70.2% of the target sample size (*n* = 5,737). To ensure a more stringent conclusion, we set the significance threshold at *p* < 0.01. The 95% CI from the adjusted GLMs indicated that individuals engaged in MVPA following a weekend warrior pattern performed significantly better than those in the inactive group when the significance threshold was set at *p* < 0.05. However, when the threshold was adjusted to *p* < 0.01, the significance disappeared. Currently, no studies have sufficient statistical power to definitively determine the training effects of the weekend warrior approach on cardiovascular fitness. Large-scale, long-term randomized controlled trials are warranted to confirm this speculation.

With respect to body composition, recent evidence has suggested that weekend warriors are associated with lower overall fatness than inactive warriors [19, 34]. However, a more complete picture of body composition, which is the proportion of body fat and fat-free mass, has rarely been studied. To address this research gap, we used the fat mass-to-fat-free mass ratio [28] to evaluate the body composition. Our findings revealed that both weekend warriors and regularly active patterns significantly lowered the fat mass-to-fat-free mass ratio, indicating that individuals following these patterns have a greater proportion of fat-free mass (lean mass and bone mass) relative to fat mass. A recent review revealed that even one weekly session of resistance exercise can increase muscle size and strength [35]. In our study, we not only confirmed the benefits of the weekend warrior pattern for body composition but also reported that these individuals performed better in muscular strength, muscular endurance, and flexibility than inactive individuals.

### Insufficiently active individuals with only 1 to 2 sessions or half the recommended activity volume on health-related physical fitness components (The secondary study’s objective)

The most commonly reported barrier to being physically active is a lack of time [3]. Although the weekend warrior activity pattern can lower the frequency commitment, it may still pose challenges for individuals who struggle to find 150 min to conduct MVPA throughout the week. For individuals who do not have sufficient time to commit 150 min of MVPA weekly, the WHO suggests that “some physical activity is better than none” [1]. However, the minimum effective dose required to yield significant fitness improvement remains unclear and undefined. Therefore, as outlined in the secondary objective, we compared the health-related fitness among the insufficiently active individuals engaging in just 1 to 2 sessions weekly and only half the recommended activity volume with the other activity groups. In our study, we adopted the data analysis methods developed by O’Donovan and colleagues [12], which categorize insufficiently active groups into low-frequency (1–2 MVPA sessions per week) and high-frequency (≥ 3 MVPA sessions per week) groups. Our adjusted GLM analyses demonstrated that insufficiently active individuals who engage in at least three days of MVPA showed better performance in most health-related fitness components (cardiorespiratory fitness, muscular strength, muscular endurance, and body composition) than inactive individuals. However, we did not observe a similar association among insufficiently active individuals who engaged in only 1–2 days of MVPA. Furthermore, we divided the duration of MVPA for insufficiently active adults into 10–74.9 min and 75–149.9 min. Our data revealed that less than half of the WHO recommended MVPA volume (i.e., fewer than 75 min) was not associated with better cardiorespiratory fitness, muscular strength, muscular endurance, and body composition than inactive individuals. Collectively, our study indicated that even individuals with limited time can still benefit all health-related fitness components by spreading them over more than three days and achieving at least a half dose of the recommended MVPA.

To further examine MVPA duration, we observed a clear dose-response relationship between weekly MVPA duration and all components of health-related fitness. Our findings align with the WHO’s recommendation that “more physical activity is better,” as higher volumes of aerobic physical activity are associated with additional health benefits, such as reduced all-cause mortality, cardiovascular disease mortality, and cancer incidence [1]. However, knowledge of the dose-response relationship between MVPA duration and health-related fitness components remains limited. Our study demonstrated that the dose-response relationship for better performance in most health-related fitness components (except flexibility) began at the 75–149.9 min per week of MVPA and continued up to 300 or more minutes per week. These results may supplement current physical activity guidelines by suggesting that more physical activity not only leads to better health outcomes but also significantly enhances health-related fitness.

### Vigorous-intensity weekend warrior activities on health-related physical fitness (The third study’s Objective)

The third objective of this study is to examine the vigorous-intensity weekend warrior activity pattern on physical fitness. Our previous pilot randomized controlled trial revealed that vigorous-intensity aerobic training with the weekend warrior approach could improve VO2max, but such improvement was not observed in the moderate-intensity weekend warrior aerobic exercise group [36]. Based on this observation, we further analyzed the relationship between the vigorous-intensity weekend warrior pattern and VO2max, along with other fitness components. We found that the vigorous weekend warrior group had a greater VO2max than the inactive group, but this advantage was not observed in the MVPA weekend warrior group. For other health-related fitness components (muscular strength, muscular endurance, flexibility, and body composition), the vigorous weekend warrior group performed better than inactive individuals. However, our questionnaire (see supplementary 1) measured only MVPA and VPA, excluding moderate-intensity activity alone. Future research should further compare moderate- and vigorous-intensity weekend warrior patterns. Additionally, our previous randomized controlled trial revealed that engaging in one session of high-intensity interval training weekly could improve cardiovascular fitness, increase fat-free mass, and reduce body adiposity, even with the weekly exercise duration below the WHO recommendation (i.e., 75 min of VPA weekly) [37]. Conversely, our fully adjusted GLM analysis revealed that, compared with inactive individuals, insufficiently active VPA patterns (less than 75 min weekly) did not significantly affect cardiorespiratory fitness or body composition. Notably, the 99% CI for body composition among the insufficiently active VPA group (−0.01, 0.01) was close to the threshold of statistical significance compared with the inactive group in the fully adjusted GLM. Therefore, additional large-scale randomized controlled trials are warranted to confirm the beneficial or non-beneficial effects of weekend warrior activity on body composition.

### Strengths of this study

There are three strengths in the methodology of this study. First, we did not use convenience sampling, which can lead to biased results. We used a random sampling method that considered the proportions of various demographic factors (age group, gender, employment status, industry, and living district) based on data from the Hong Kong Government’s 2021 Population Census. Second, to increase reliability when measuring MVPA, we incorporated the RPE scale and provided multiple examples to describe MVPA (see Supplementary 1). Finally, to the best of our knowledge, this is the first study to examine the associations between the weekend warrior activity pattern and all five health-related fitness components. These findings may further our understanding of the development of physical activity strategies for maintaining or promoting health-related physical fitness.

### Limitation of this study

Our study has several limitations. First, the cross-sectional study design could not demonstrate causal effects and provide balanced sample size of each physical activity group. Therefore, additional randomized controlled trials are needed to investigate the effectiveness of training on physical fitness, adherence to weekend warrior training, and the occurrence of overuse injuries. Second, our assessment of physical activity patterns was based on self-report questionnaires. Future research should integrate objective assessments, such as accelerometers. Third, it is worth noting that our questionnaire (supplementary 1) measured only MVPA and VPA, and the associations of light-intensity and moderate-intensity activity patterns with physical fitness remain unknown. Fourth, our survey was planned before the WHO updated the physical activity guidelines in 2020. The 2010 version [38] we used in the present study only measured activity bouts lasting at least 10 min, whereas the current guidelines consider that any activity duration should be considered [1]. Finally, field-based measurements were used to assess VO2max, muscular strength, and body composition. Future studies may consider using the gold-standard measurement, such as gaseous analysis for VO2max, isokinetic dynamometry for muscular strength, and dual-energy X-ray absorptiometry scans for body composition evaluation.

## Conclusion

Our study reveals that (1) the weekend warrior MVPA pattern benefits most health-related fitness components, except cardiovascular fitness; (2) even when falling below the recommended MVPA volume, participating in more than three sessions per week or achieving at least half of the recommended MVPA dose is associated with better health-related fitness compared to inactivity; and (3) engaging in vigorous-intensity weekend warrior activity could be associated with better performance in all health-related fitness components. These findings offer valuable insights for shaping future public health guidelines, particularly for individuals with busy schedules. To further validate these results, randomized controlled trials should be conducted.

## Supplementary Information

Supplementary Material 1.

## Data Availability

The data are available from the corresponding author upon reasonable request and approval from the Leisure and Cultural Services Department, The Government of Hong Kong.
