# Supplementary material for: Association of “weekend warrior” and leisure time physical activity patterns with health-related physical fitness: a cross-sectional study
Source: BMC Public Health. 2025 Oct 3;25:3316. doi: 10.1186/s12889-025-24711-y (PMC12495872; doi:10.1186/s12889-025-24711-y)
Supplement: Supplementary file 1 — Supplementary Material 1. [file 12889_2025_24711_MOESM1_ESM.docx]

**Supplementary 1. Questionnaire for MVPA and VPA**

Q18 In the recent past year, on how many days did you do vigorous intensity physical activities for at least 10 minutes at a time in an average week? Vigorous intensity physical activities cause feeling of being exhausted. They also induce significantly rapid breathing and profuse sweating. You find difficult to talk to others when doing the activities. Vigorous intensity physical activities should be in similar intensity level with running or removing heavy weights of 10 kg (such as 20 lunch boxes, 5 bottles of soft drinks in 2 liters).

Other examples of vigorous intensity physical activity are playing ball games (such as basketball, soccer, single tennis), continuous swimming (excluding slow swimming), fast and continuous ice-skating, rope skipping, uphill climbing, non-stop walking upstairs, aerobic dance, fast cycling, judo, taekwondo, rock climbing. In the Rating of Perceived Exertion (RPE), vigorous intensity physical activities are scored 8-9 meaning the intensity between very strong and extremely strong.

|  | 0 day 0 | 🡪【**skip to Q20**】 |
| --- | --- | --- |
|  | 1 day 1 | 5 days 5 |
|  | 2 days 2 | 6 days 6 |
|  | 3 days 3 | 7 days 7 |
|  | 4 days 4 |  |

Q19 **[Answered only by those did vigorous intensity physical activities for at least 10 minutes at a time in an average week (i.e. Q18≠0)]** How much time did you spend on vigorous intensity physical activities in a week?

|  |  |  |  | minutes |
| --- | --- | --- | --- | --- |

Q20 In the recent past year, on how many days did you do moderate or above including vigorous intensity physical activities for at least 10 minutes at a time in an average week? Moderate intensity physical activities cause little feeling of being fatigued. They also induce quicker-than-normal breathing and little sweating. You find difficult to croon when doing the activities. Moderate intensity physical activities should be in similar intensity level with fast walk or walking with weights of 4.5-9kg carried (such as a heavy schoolbag, 2 packs of A-4 size paper, 2-4 bottles of soft drink in 2 liters, 24 cans of soft drink).

Other examples of moderate intensity physical activity are playing ball games (such as baseball, softball, badminton, volleyball, table-tennis, double tennis), downhill climbing, swimming in normal speed, cycling in normal speed, non-stop walking downstairs, dancing (such as Hip Hop, Social dance, Ballet, Folk), skateboarding, horizontal bar gymnastics, playing frisbee, hard cleansing work (such as removing desks and chairs in classroom, floor cleansing by hand, window cleansing). In the Rating of Perceived Exertion (RPE), moderate intensity physical activities are scored 4-7 meaning the intensity over moderate and up to very strong.

|  | 0 day 0 | 🡪【**skip to Q22**】 |
| --- | --- | --- |
|  | 1 day 1 | 5 days 5 |
|  | 2 days 2 | 6 days 6 |
|  | 3 days 3 | 7 days 7 |
|  | 4 days 4 |  |

**[Logic check: Response of Q20 should not be less than response of Q18]**

Q21 **【Answered only by those did moderate or above including vigorous intensity physical activities for at least 10 minutes at a time in an average week (i.e. Q20≠0)】**How much time did you spend on moderate or above including vigorous intensity physical activities in a week?

|  |  |  |  | minutes |
| --- | --- | --- | --- | --- |

**【Logic check: Response of Q21 should not be less than response of Q19】**
